# Supplementary material for: Incomplete influenza A virus genomes occur frequently but are readily complemented during localized viral spread
Source: Nat Commun. 2019 Aug 6;10:3526. doi: 10.1038/s41467-019-11428-x (PMC6684657; doi:10.1038/s41467-019-11428-x)
Supplement: Supplementary file 1 — Supplementary Information [file 41467_2019_11428_MOESM1_ESM.pdf]

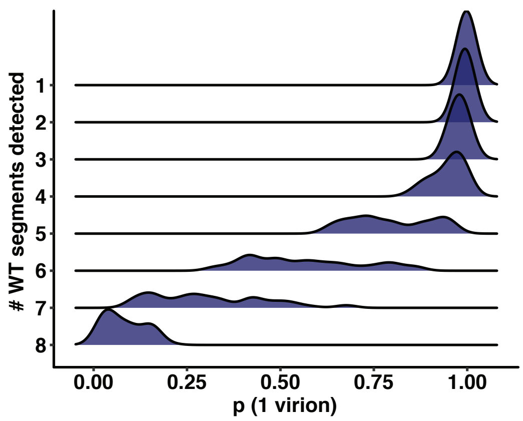

Supplementary Figure 1 — Cells containing more segments are more likely to be multiply infected.

Bayes' rule was used to calculate the probability that each cell was infected with exactly 1 virion, based on the number of infected cells in each experiment, and each cell's combination of segment presences and absences. The distribution of probabilities is shown stratified by the number of segments present per cell.

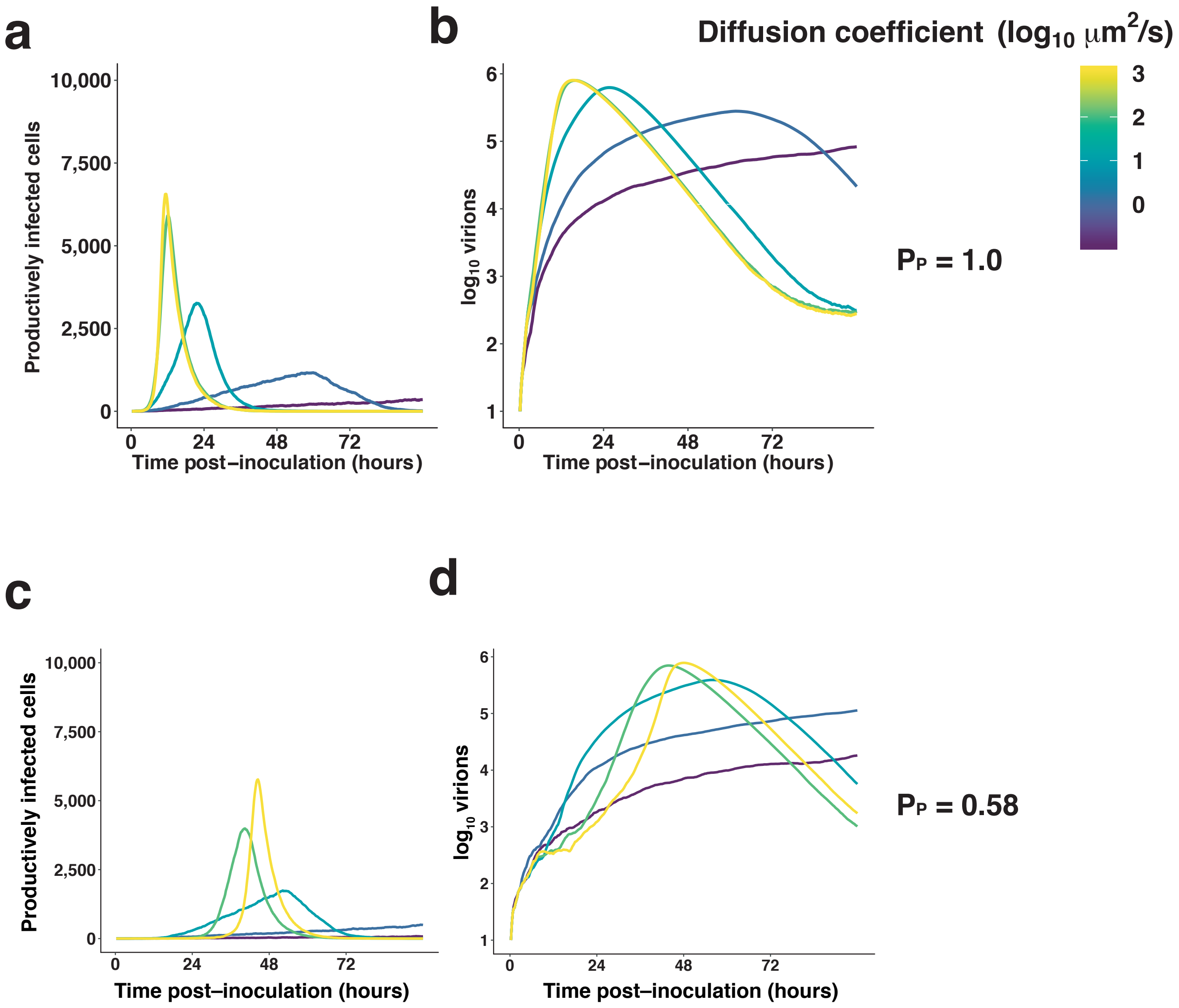

Supplementary Figure 2 — Infection dynamics are influenced by diffusion coefficient and the presence of incomplete viral genomes.

(A, B) The dynamics of infection, in terms of productively infected cells (A) or virions present (B) are shown for a virus with complete genomes (Pp = 1.0).

(C, D) The dynamics of infection, in terms of productively infected cells (C) or virions present (D) are shown for a virus with incomplete genomes (Pp = 0.58).

Lines are colored by diffusion coefficient.

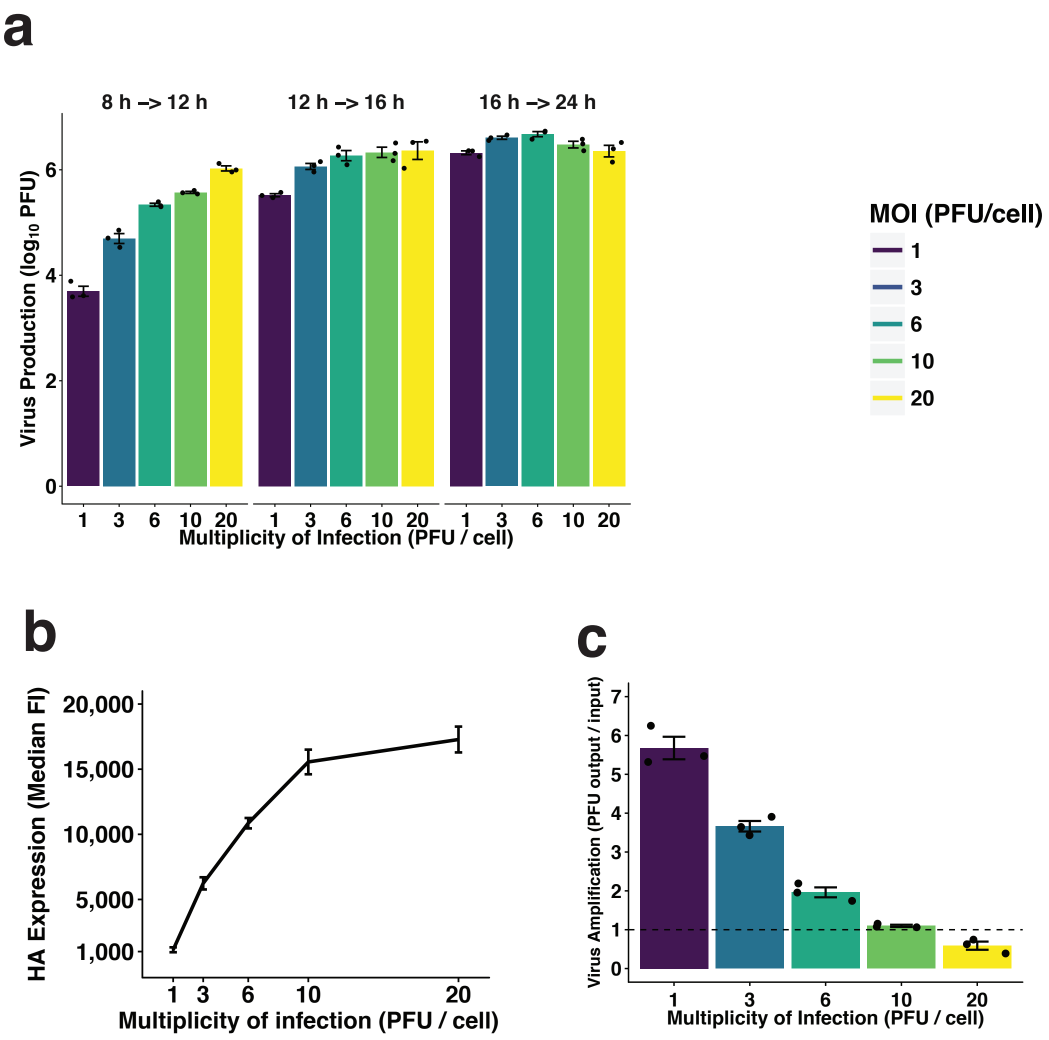

Supplementary Figure 3 — Multiple infection provides a kinetic benefit to viral replication and enhances gene expression, at the cost of reduced viral amplification.

(A) The amount of virus produced (in PFU) in three distinct time periods was calculated at each MOI.

(B) The amount of HA expression (median fluorescence intensity) among HA+ cells is shown across a range of MOIs at 12 hours post-inoculation.

(C) Amplification of virus was calculated by dividing the amount of virus produced (in PFU) after 48 hours by the number of PFU used to incoulate cells.

Error bars represent mean ± S.E.

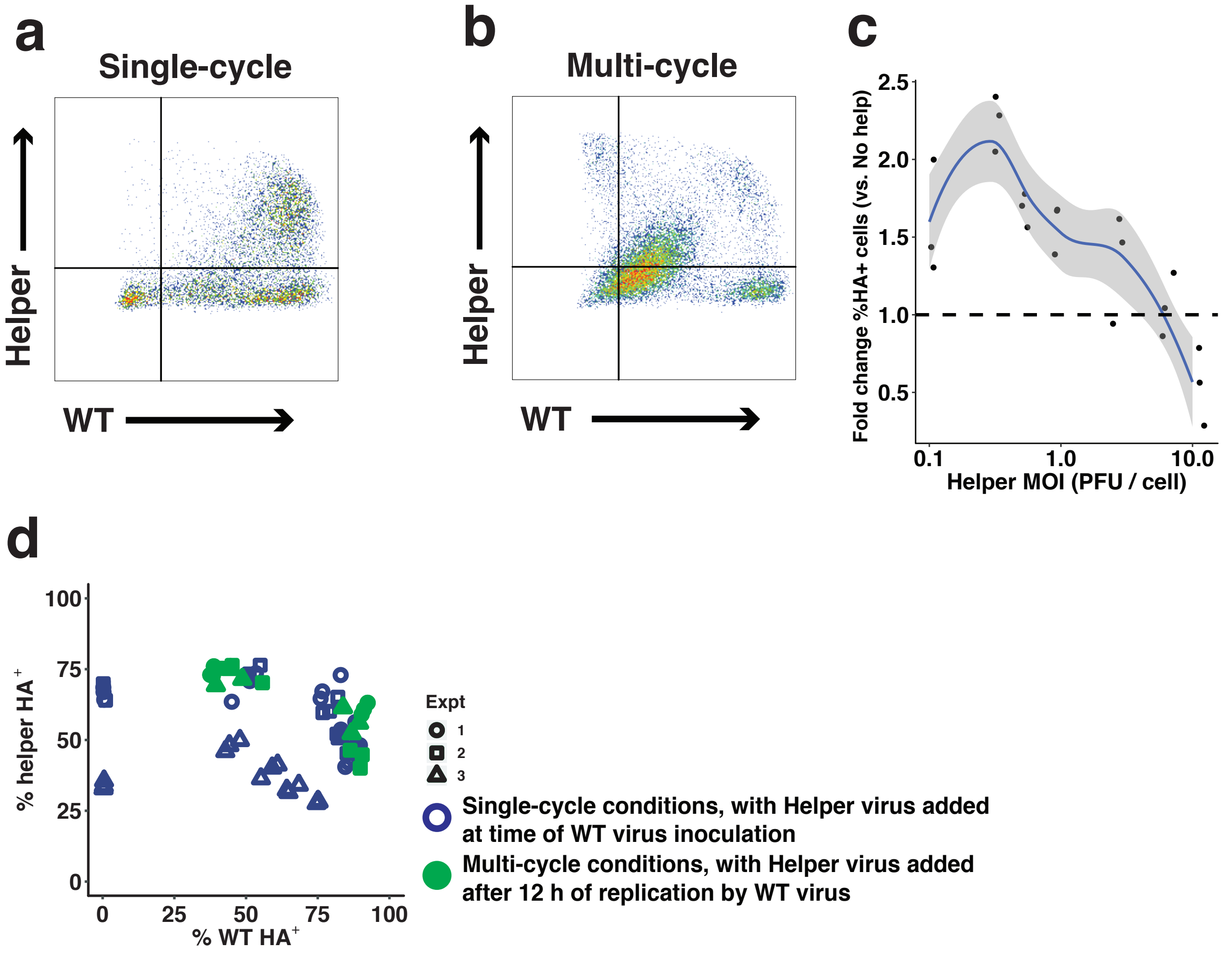

Supplementary Figure 4 — Intermediate levels of Pan/99-Helper virus enable detection of incomplete WT viral genomes, without being hindered by super-infection exclusion.

(A,B) Representative flow cytometry measurement of Pan/99-WT HA when Pan/99-Helper virus is added simultaneously (A), or following 12 h of multi-cycle replication (B). Pan/99-WT MOI = 0.1 PFU/cell for simultaneous co-infection, 0.01 PFU/cell for multi-cycle replication. Pan/99-Helper virus MOI was 0.3 PFU/cell in both cases.

(C) Cells were inoculated with Pan/99-WT (MOI = 0.01 PFU/cell) and Pan/99-Helper at a range of MOI, then incubated under single-cycle conditions before staining for expression of WT and Helper HA proteins. The extent to which Pan/99-Helper increased numbers of WT HA+ cells (relative to controls infected with only Pan/99-WT) was calculated at each Pan/99-Helper MOI. Curve and ribbon represent mean and 95% confidence interval (mean ± 1.96 * S.E.), respectively, of local regression.

(D) Pan/99-Helper virus HA expression was plotted against Pan/99-WT virus HA expression to determine whether mutli-cycle replication of WT virus resulted in super-infection exclusion of Helper virus, thereby limiting the ability of Helper virus to complement IVGs after multi-cycle replication. For single-cycle conditions, cells were simultaneously inoculated with Pan/99-WT virus and Pan/99-Helper, then incubated under single-cycle conditions for 12 h (blue open circles). For multi-cycle conditions, cells were inoculated with Pan/99-WT virus at low MOI and incubated under multi-cycle conditions for 12 h, then inoculated with Pan/99-Helper virus and incubated under single-cycle conditions for 12 h (green open circles).

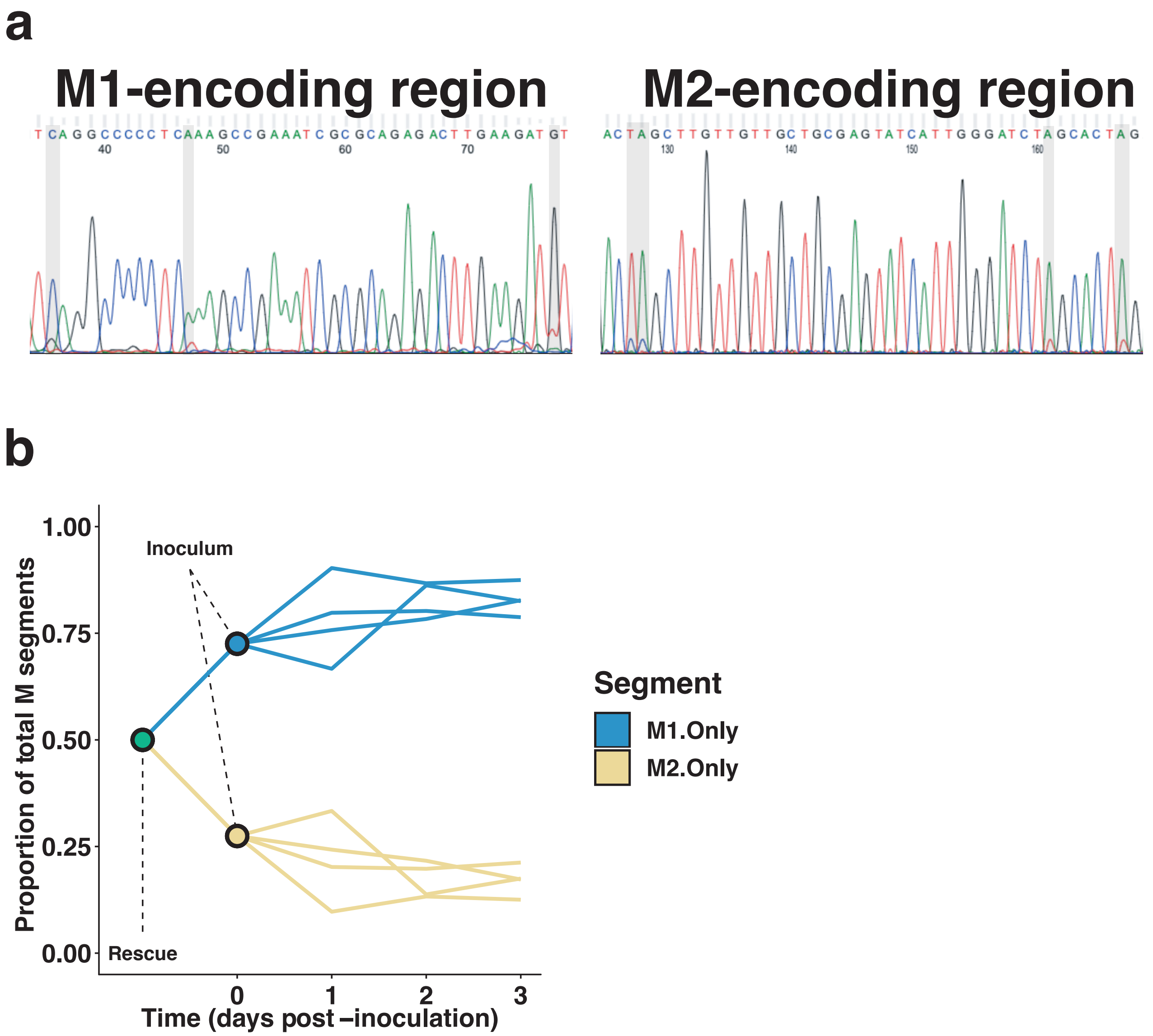

Supplementary Figure 5 — Both M1.Only and M2.Only genome segments are maintained in vivo following infection with Pan/99-M.STOP virus.

(A) M segments of virus recovered from guinea pigs at 2 d post-inoculation with Pan/99-M.STOP virus were amplified by PCR, and the M1-encoding (left) and M2-encoding (right) regions were sequenced to verify maintenance of both alleles in vivo. Shaded gray rectangles highlight mutated regions.

(B) Guinea pigs were inoculated with Pan/99-M.STOP virus, and ddPCR was used to quantify copy numbers of M1.Only (blue) and M2.Only (yellow) segments, expressed as a proportion of all M segments. Blue and yellow dots represent the frequencies of M1.Only and M2.Only segments present in the virus stocks used to prepare inocula. The green dot represents the plasmid mixture used to generate the virus, in which equal molar quantities of M1.Only and M2.Only pDP plasmids were combined.

Supplementary Table 1 — Genotype of Pan/99-Helper virus

Segment	Mutations relative to Pan/99-WT
PB2	A550C, G552A, A555C, C556T, A617C, T621C, T622A, C623G
PB1	C346T, T348G, A351G, T441A, T444A, A447T
PA	G603A, T604A, C605G, C747T, T750C, G753A
HA	T308C, C311A, C313T, A464T, C467G, T470A
NP	C537T, T538A, C539G, G606C, A609T, G615C
NA	C418G, T421A, A424C, T511A, T514A, A517G
M	C413T, C415G, A418C, A517G, G523A, A526C
NS	A210C, G212A, G215A, T218C, C329T, C335T, A341G

Supplementary Table 2 — Primers for single-cell assay

Primer Name	Sequence (5' — 3')	Virus targeted
PB2 537F wt	TGAAGTGGGAGCCAGGATAC	Pan/99-WT
PB2 640R wt	ATGCAACCATCAAGGGAGAA	Pan/99-WT
PB1 332F wt	TTGAGAGCTCATGCCTTGAA	Pan/99-WT
PB1 459R wt	GTTGGCTAATGCAGTTGCTG	Pan/99-WT
PA 595F wt	TTTCGTCAGTCCGAAAGAGG	Pan/99-WT
PA 741R wt	AGCTTGCCCTCAATGCAGCCG	Pan/99-WT
HA 266F wt	ACCCTCATTGTGATGGCTTC	Pan/99-WT
HA 452R wt	GTTCCATTCTGAGCGACTCC	Pan/99-WT
NP 520F wt	ATGGATCCCAGAATGTGCTC	Pan/99-WT
NP 625R wt	TCAGCTCCATCACCATTGTC	Pan/99-WT
NA 408F wt	ATCAATTTGCCCTTGGACAG	Pan/99-WT
NA 528R wt	CCCAAATGAAATGGAACACC	Pan/99-WT
M 402F wt	GTTGCATGGGCCTCATATAC	Pan/99-WT
M 535R wt	ATTGGTTGTTGCCACCATTTG	Pan/99-WT
NS 173F wt	CCATGTTGGAAAGCAGATTG	Pan/99-WT
NS 321R wt	GGGCATTAGCATGAACCAGT	Pan/99-WT
PB2 537F var	TGAAGTGGGAGCCCGAATCT	Pan/99-Helper
PB2 640R var	ATGCAACCATCAACGGACTG	Pan/99-Helper
PB1 332F var	TTGAGAGCTCATGCTTGGAG	Pan/99-Helper
PB1 459R var	GTTGGCTAATGCTGTAGCAG	Pan/99-Helper
UnivF(A) + 6	GCGCGCAGCAAAAGCAGG	Pan/99-WT and Pan/99-Helper
UnivF(G) + 6	GCGCGCAGCGAAAGCAGG	Pan/99-WT and Pan/99-Helper

Supplementary Table 3 — Primers and probes for ddPCR

Primer/Probe Name	Sequence (5' — 3')
M2 F ddPCR Primer	ACTCATCCTAGCTCCAG
M2 R ddPCR Primer	CCGTGTTTGAAGAGTCG
M2.Only ddPCR Probe (M2 WT)	HEX – CCATTCGTTTCTGATAGGTCTG – BHQ1
M1.Only ddPCR Probe (M2 Mutant)	6-FAM – CCATACGCTTCTGGTACGTCTG – BHQ1
M1 Sequencing Primer	TAGATATTGAAAGATGAGCC
M2 Sequencing Primer	TAGATATTGAAAGATGAGCC
NS F ddPCR Primer	ACCTGCTTCGCGATACATAAC
NS R ddPCR Primer	AGGGGTCCTTCCACTTTTTG
NS ddPCR Probe	6-FAM – AGAAACTGGTTCATGCTAATGCCCA – BHQ1
