# Supplementary material for: Incomplete influenza A virus genomes occur frequently but are readily complemented during localized viral spread
Source: Nat Commun. 2019 Aug 6;10:3526. doi: 10.1038/s41467-019-11428-x (PMC6684657; doi:10.1038/s41467-019-11428-x)
Supplement: Supplementary file 3 — Reporting Summary [file 41467_2019_11428_MOESM3_ESM.pdf]

nature research

Corresponding author(s): Anice C. Lowen

Last updated by author(s): Jan 7, 2019

Reporting Summary

Nature Research wishes to improve the reproducibility of the work that we publish. This form provides structure for consistency and transparency in reporting. For further information on Nature Research policies, see Authors & Referees and the Editorial Policy Checklist.

Statistics

For all statistical analyses, confirm that the following items are present in the figure legend, table legend, main text, or Methods section.

n/a	Confirmed	
☐	☒	The exact sample size (*n*) for each experimental group/condition, given as a discrete number and unit of measurement
☐	☒	A statement on whether measurements were taken from distinct samples or whether the same sample was measured repeatedly
☐	☒	The statistical test(s) used AND whether they are one- or two-sided<br>*Only common tests should be described solely by name; describe more complex techniques in the Methods section.*
☐	☒	A description of all covariates tested
☐	☒	A description of any assumptions or corrections, such as tests of normality and adjustment for multiple comparisons
☐	☒	A full description of the statistical parameters including central tendency (e.g. means) or other basic estimates (e.g. regression coefficient) AND variation (e.g. standard deviation) or associated estimates of uncertainty (e.g. confidence intervals)
☐	☒	For null hypothesis testing, the test statistic (e.g. *F*, *t*, *r*) with confidence intervals, effect sizes, degrees of freedom and *P* value noted<br>*Give P values as exact values whenever suitable.*
☒	☐	For Bayesian analysis, information on the choice of priors and Markov chain Monte Carlo settings
☒	☐	For hierarchical and complex designs, identification of the appropriate level for tests and full reporting of outcomes
☐	☒	Estimates of effect sizes (e.g. Cohen's *d*, Pearson's *r*), indicating how they were calculated

Our web collection on statistics for biologists contains articles on many of the points above.

Software and code

Policy information about availability of computer code

Data collection	Custom code, generated in R, was used to collect data on i) the theoretical impact of incomplete genomes for viral infectivity at single cell and population levels, and ii) to test whether spatially structured viral spread is expected to mitigate the costs of incomplete viral genomes.
Data analysis	Custom code described initially by Fonville et al. (PLOS Pathogens, 2015) was adapted to R and used to evaluate whether PP values estimated herein are consistent with previously observed levels of reassortment (Figure 1b). Custom code, generated in R, was used to analyze the rest of the raw data.<br>All code used in generating simulations results, analysis, and visualizations can be found at the following URL: https://github.com/njacobs627/Pan99_IVGs_Spatial_Structure.

For manuscripts utilizing custom algorithms or software that are central to the research but not yet described in published literature, software must be made available to editors/reviewers. We strongly encourage code deposition in a community repository (e.g. GitHub). See the Nature Research guidelines for submitting code & software for further information.

Data

Policy information about availability of data

All manuscripts must include a data availability statement. This statement should provide the following information, where applicable:

- Accession codes, unique identifiers, or web links for publicly available datasets
- A list of figures that have associated raw data
- A description of any restrictions on data availability

All raw data is provided as a Source Data file, and is available at the following URL: https://github.com/njacobs627/Pan99_IVGs_Spatial_Structure.

October 2018

Field-specific reporting

Please select the one below that is the best fit for your research. If you are not sure, read the appropriate sections before making your selection.

☒ Life sciences ☐ Behavioural & social sciences ☐ Ecological, evolutionary & environmental sciences

For a reference copy of the document with all sections, see nature.com/documents/nr-reporting-summary-flat.pdf

Life sciences study design

All studies must disclose on these points even when the disclosure is negative.

Sample size	Sample size for animal experiments (n=4 per virus, per dose) was selected based on variance observed in prior experiments of a similar nature, as well as practical considerations. For determination of infectious dose, where a large number of doses are tested, practical considerations include cost of animals and space constraints. For evaluating transmission, space constraints within environmental chambers limit group sizes to four infected plus four exposed animals for each virus tested.
Data exclusions	No data were excluded from analysis.
Replication	Reproducibility of PP (probability present) values for influenza A virus genome segments was verified by performing multiple independent experiments, as indicated in Figure 1. The probabilistic model used to predict effects of incomplete genomes on infectivity (reported in Figures 2 and 3) was validated by comparing results of the analytic derivation to those of a Monte Carlo simulation. Single cycle growth data shown in Figure 5 are representative of two independent experiments. Three independent experiments were performed to monitor incomplete viral genome levels under differing conditions of spatial structure (Figure 6). Results were consistent among experiments. Infectivity of the M.STOP virus relative to WT was examined in multiple independent systems (Figure 7). Similar patterns of M.STOP virus growth kinetics in guinea pigs was observed in independent experiments (results shown in Figures 8A and 8B). No attempt to replicate transmission data was made.
Randomization	No formal randomization method was used.
Blinding	Investigators were not blinded to group allocation.

Reporting for specific materials, systems and methods

We require information from authors about some types of materials, experimental systems and methods used in many studies. Here, indicate whether each material, system or method listed is relevant to your study. If you are not sure if a list item applies to your research, read the appropriate section before selecting a response.

Materials & experimental systems

n/a	Involved in the study	
☐	☒	Antibodies
☐	☒	Eukaryotic cell lines
☒	☐	Palaeontology
☐	☒	Animals and other organisms
☒	☐	Human research participants
☒	☐	Clinical data

Methods

n/a	Involved in the study	
☒	☐	ChIP-seq
☐	☒	Flow cytometry
☒	☐	MRI-based neuroimaging

Antibodies

Antibodies used	His-Alexa 647, clone ABD2.2.20, Qiagen, catalog no. 35370, Lot # 160014628<br>HA.tag FITC, clone HA-7, Sigma, catalog no. H7411-100UG, Lot # 086M4842V<br>anti-M1, clone GA2B (ThermoFisher MA1-80736), was conjugated to Pacific Blue using conjugation kit from ThermoFisher (P30013), Lot # TG2603994<br>anti-M2, clone 14C2, PE-conjugated, Santa Cruz, catalog no. sc-32238 PE, Lot #B1717
Validation	Qiagen states that the His-Alexa 647 antibody showed negligible cross reactivity with crude E. coli, yeast, mammalian, or insect cell lysates. Specificity of all four antibodies for cells infected with viruses encoding the relevant epitopes was verified by staining uninfected cells as a negative control in all flow experiments. Lack of reactivity of anti-HIS for HA and of anti-HA for HIS was also verified by reciprocal staining of cells infected with HIS-only or HA-only encoding viruses. Recognition of M1 and M2 proteins by the respective antibodies has been verified in-house by Western blotting.

Eukaryotic cell lines

Policy information about cell lines

Cell line source(s)	Madin-Darby canine kidney (MDCK) cells were obtained from Peter Palese, Icahn School of Medicine at Mount Sinai<br>293T cells (CRL-3216) were obtained from ATCC
Authentication	Cell lines used were not authenticated
Mycoplasma contamination	Cell lines tested negative for mycoplasma
Commonly misidentified lines<br>(See ICLAC register)	No commonly misidentified lines were used

Animals and other organisms

Policy information about studies involving animals; ARRIVE guidelines recommended for reporting animal research

Laboratory animals	Female, Hartley strain guinea pigs (Cavia Porcellus), 4-6 weeks of age, were obtained from Charles River Laboratories
Wild animals	No wild animals were used.
Field-collected samples	No field-collected samples were used.
Ethics oversight	Animal studies were reviewed and approved by the Institutional Animal Care and Use Committee of Emory University.

Note that full information on the approval of the study protocol must also be provided in the manuscript.

Flow Cytometry

Plots

Confirm that:

☒ The axis labels state the marker and fluorochrome used (e.g. CD4-FITC).

☒ The axis scales are clearly visible. Include numbers along axes only for bottom left plot of group (a 'group' is an analysis of identical markers).

☒ All plots are contour plots with outliers or pseudocolor plots.

☒ A numerical value for number of cells or percentage (with statistics) is provided.

Methodology

Sample preparation	As described in Methods, cells were treated with trypsin-EDTA to remove them from plates, then resuspended in FACS buffer (PBS + 2% FCS) and spun at 2500 rpm for 5 minutes. Cells were resuspended in FACS buffer and transferred to V-bottom plates, spun at 2,500 rpm for 5 minutes, then resuspended in 50 uL FACS buffer containing staining antibodies. After staining at 4 C for 30 minutes, cells were washed three times by resuspension in FACS buffer and centrifugation. After the final wash, cells were resuspended in FACS buffer containing 7-AAD live/dead stain and analyzed on a BD Fortessa.
Instrument	BD Fortessa was used for analysis, and BD Aria II was used for sorting.
Software	Data were analyzed in FlowJo v. 10.1
Cell population abundance	N/A
Gating strategy	1.) SSC vs. FSC gating to exclude debris. 2.) FSC-H vs. FSC-A gating to exclude doublets.<br>3.) 7-AAD vs. FSC-A gating to exclude dead cells. 4.) Alexa 647 (His) vs. FITC (HA) gating to quantify His+ and HA+ cells.<br>Boundaries for Gate 4 were based on a PBS-inoculated control.

☒ Tick this box to confirm that a figure exemplifying the gating strategy is provided in the Supplementary Information.
